# Macrolide versus Non-Macrolide in Combination with Steroids for the Treatment of Lobar or Segmental *Mycoplasma pneumoniae* Pneumonia Unresponsive to Initial Macrolide Monotherapy

**DOI:** 10.3390/antibiotics11091233

**Published:** 2022-09-10

**Authors:** Eunha Bae, Ye Ji Kim, Hyun Mi Kang, Dae Chul Jeong, Jin Han Kang

**Affiliations:** 1 Department of Pediatrics, College of Medicine, The Catholic University of Korea, Seoul 06591, Korea; 2 Vaccine Bio Research Institute, College of Medicine, The Catholic University of Korea, Seoul 06591, Korea

**Keywords:** macrolide, *Mycoplasma pneumoniae*, resistance

## Abstract

In the last few decades, macrolide-resistant *Mycoplasma pneumoniae* (MRMP) has been increasing in proportion. This study aimed to evaluate the treatment outcomes of children with lobar or segmental MP pneumonia unresponsive to the initial 3–5-day macrolide therapy, who then switched to either a non-macrolide, macrolide + steroid, or a non-macrolide + steroid regimen, according to the 2019 KSPID and KAPARD guideline during the 2019–2020 Mycoplasma epidemic in South Korea. A total of 190 patients <18 years old were admitted during the study period for MP lobar or segmental pneumonia, and 16.8% (*n* = 32/190) were responsive to the initial macrolide monotherapy, whereas 83.2% (158/190) were refractory. The median age of the patients was 7 (interquartile range [IQR], 5–9) years old and 46.2% (*n* = 73/158) were male. The overall treatment success rates of non-macrolide, macrolide + steroid, and non-macrolide + steroid groups were 46.2%, 80.8%, and 100.0%, respectively. Patients in the non-macrolide + steroid group had the shortest fever duration after a regimen change of 1 (IQR, 0–3) day compared with patients in the non-macrolide group and macrolide + steroid group; 2 (IQR, 1–4) days and 2 (IQR, 1–3.3) days (*p* = 0.004), respectively. Follow-up CRP (ß, 0.169; CI, 0.050–0.287; *p* = 0.006), macrolide + steroid therapy (ß, −1.694; CI, −2.463–−0.925; *p* < 0.001), and non-macrolide+ steroid therapy (ß, −2.224; CI, −3.321–−1.127; *p* < 0.001) were shown to be significantly associated with the duration of fever after admission. To conclude, in patients with severe MP pneumonia that failed to respond to the initial macrolide therapy, a non-macrolide + steroid had the highest treatment success rate and a shorter duration of fever.

## 1. Introduction

*Mycoplasma pneumoniae* (MP) is one of the most common causes of bacterial pneumonia in children and adolescents [1]. MP is a tiny pleomorphic bacterium and is the smallest self-replicating bacteria pathogenic in humans, its only known host. In children over five years old, 40% of community-acquired pneumonia is caused by MP and about 18% requires hospitalization [2]. MP epidemics occur at an interval of 4–7 years due to waning herd immunity and the introduction of new subtypes into the population [3]. Because MP lacks a cell wall, beta-lactam antibiotics are known to be ineffective, and the use of macrolides has been known to decrease the duration of illness [4].

In the last few decades, macrolide-resistant (MR) MP has been increasing in proportion, leading to children becoming unresponsive to the initial macrolide therapy [5]. Therefore, even considering the potential toxicities of tetracyclines and fluoroquinolones in children, their uses have been increasing in those suspected of MRMP or in patients with clinical deterioration [6,7]. Because immunologic mechanisms are known to play an important role in MP, steroids have also been used effectively and safely in children with severe pneumonia [8,9].

For patients who are unresponsive to the initial macrolide therapy and are diagnosed with severe pneumonia, the 2019 guideline from the Korean Society of Pediatric Infectious Diseases (KSPID) and the Korean Academy of Pediatric Allergy and Respiratory Disease (KAPARD) recommends: (1) switching to non-macrolide, such as quinolones or tetracyclines; (2) the addition of steroids in combination with a macrolide, or (3) the addition of steroids in combination with a non-macrolide. To date, there has been a paucity of data on which alternative treatment is more effective in children with severe MP pneumonia upon lack of response or disease progression despite macrolide therapy. Therefore, the purpose of this study was to evaluate the outcomes of children with lobar or segmental MP pneumonia that is unresponsive to initial macrolide therapy, who received (1) non-macrolide, (2) macrolide + steroids, or (3) non-macrolide + steroids in accordance with the 2019 guideline during the 2019–2020 Mycoplasma epidemic in South Korea [10].

## 2. Materials and Methods

### 2.1. Study Population

This was a retrospective cohort study of children below 18 years old, admitted during the 2019–2020 MP outbreak for lobar or segmental MP pneumonia at a tertiary referral university hospital located in Seoul, Korea.

The inclusion criteria for study participants were as follows: (1) diagnosed with lobar or segmental MP pneumonia, (2) initially treated with a macrolide, and (3) remained unresponsive to macrolide after more than three days of administration. These patients were considered ‘macrolide-refractory severe mycoplasma pneumonia’ and were included in the study for further analysis.

The exclusion criteria were as follows: (1) underlying immunocompromising disease, (2) administration of immunosuppressants prior to admission, (3) initial use of steroids, tetracyclines, or quinolones upon MP pneumonia diagnosis, (4) co-infection with another virus or bacteria, (5) diagnosed with pneumonia of another etiology within one month of MP pneumonia diagnosis, and (6) cause of pneumonia other than MP.

All X-rays were read by a pediatric radiologist, and the readings were collected and reviewed. The following parameters were reviewed from the electronic medical records of the patients included in the study: sex, age at admission, body weight, chest radiographic readings, admission and discharge date, and duration of fever prior to and after admission. The following data were collected from the electronic medical records of the patients: MP PCR results, Mycoplasma IgM, Mycoplasma IgG, initial and follow-up complete blood cell count (CBC), c-reactive protein (CRP), erythrocyte sedimentation rate (ESR), aspartate transaminase (AST), alanine transaminase (ALT), and lactate dehydrogenase (LDH), blood culture results, administered drugs and dosages, and treatment response.

### 2.2. Study Design and Definitions

Patients who were considered to have ‘macrolide-refractory severe MP pneumonia’ were included as study participants and were divided into three groups depending on their next treatment regimen: non-macrolide (either a tetracycline or quinolone), macrolide + steroid, and non-macrolide + steroid group. The chest X-rays, duration of admission, duration of fever, laboratory changes, and treatment responses of the three groups were assessed. All patients were given chest X-rays at initial work-up, at regimen change, and 3–4 days after regimen change to determine the treatment response.

In this study, MP pneumonia was diagnosed in patients who fulfilled all 4 criteria: (1) persistent fever ≥ 38.0 °C, (2) crackle or decreased breath sounds upon auscultation, (3) lobar or segmental consolidative lesions on chest radiographs, and (4) positive sputum or nasopharyngeal swab tested by MP PCR or film array. Treatment response was defined as defervescence (<38.0 °C for ≥24 h) within 2–3 days after initiating treatment and no aggravation in the degree of pneumonic lesions on chest radiographs. Treatment refractory was defined as ‘persistent fever’ and ‘aggravation or no change in the degree of pneumonic lesions on chest radiographs’ after 3 days of treatment. Both definitions are based on the 2019 guideline from the KSPID and KAPARD, which recommends a switch or maintenance in treatment regimen upon the fulfillment of these criteria [10]. Treatment success was determined when the final treatment regimen used brought about clinical resolution. Severe pneumonia was defined according to the 2017 guidelines for antibiotics use in children with acute lower respiratory tract infections [11].

### 2.3. Statistics

The chi-square test was used to compare categorical variables, and the Mann–Whitney U test or Kruskal–Wallis H test was used to compare non-categorical variables. The paired *t*-test was used to compare initial and follow up laboratory findings for each group. The linear regression model was used in the univariate and multivariate regression analyses to find factors associated with fever duration after admission. The logistic regression analysis was used to find the odds of fever prolonging ≥4 days after a treatment regimen switch in the three treatment groups. All tests were two sided, and a *p*-value of <0.05 was considered statistically significant.

## 3. Results

### 3.1. Study Population

During the May 2019 to March 2020 MP epidemic, a total of 190 patients were admitted for MP lobar or segmental pneumonia and were initiated with macrolide therapy. Of these, 158 patients were considered refractory to initial macrolide monotherapy, and were therefore included as study participants. The median age of the patients was 7 (interquartile range [IQR], 5–9) years old, and 46.2% (*n* = 73/158) were male. The median fever duration prior to admission was 5 (IQR, 4–6) days, and the median duration of admission was 5 (IQR, 4–6) days. A total of 18.4% (*n* = 29/158) of the study participants had bilateral lung involvement on chest X-rays. Lobar involvement was observed in 39.2% (*n* = 62/158), while 60.8% (*n* = 96/158) had segmental involvement. Pleural effusion was observed in 12.5% (*n* = 15/158) of the patients’ chest X-rays (Table 1).

**Figure 1:**
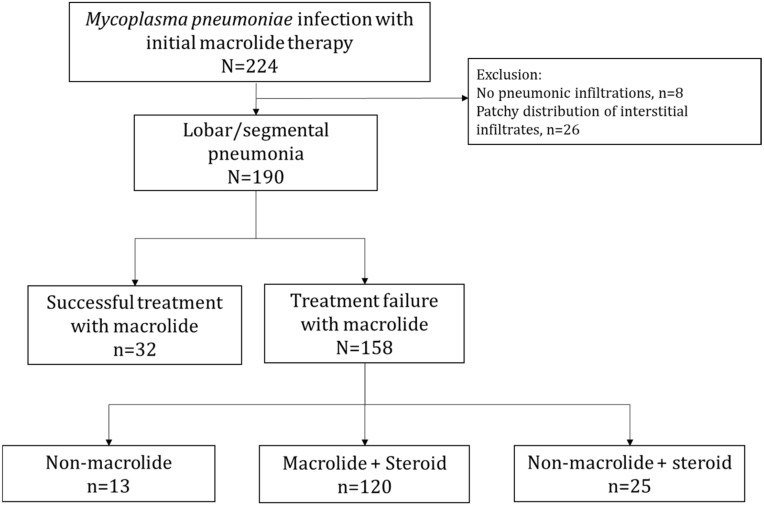
Flow chart of patients included as study participants. Patients were divided into three treatment groups depending on the alternative regimen administered after the initial macrolide therapy: 8.2% (*n* = 13/158) were switched to a non-macrolide (doxycycline, *n* = 13), 75.9% (*n* = 120/158) were administered macrolide + steroid, and 15.8% (*n* = 25/158) were switched to a non-macrolide (doxycycline, *n* = 21; levofloxacin, *n* = 4) + steroid.

Patients were divided into three treatment groups depending on the alternative regimen administered after the initial macrolide therapy: 8.2% (*n* = 13/158) were switched to a non-macrolide (doxycycline, *n* = 13), 75.9% (*n* = 120/158) were administered macrolide + steroid, and 15.8% (*n* = 25/158) were switched to a non-macrolide (doxycycline, *n* = 21; levofloxacin, *n* = 4) + steroid (Figure 1). There were no significant differences among the three groups in the median age, sex, fever duration prior to admission, or extent of the infiltrations on initial chest X-rays at admission. However, a difference was found in the fever duration after regimen change (*p* = 0.004) and admission duration (*p* = 0.010) in the three groups (Table 1). All the patients who were given a macrolide were administered either roxithromycin or clarithromycin. In the macrolide + steroid group, the median steroid dose administered in prednisolone dose equivalent was 0.8 (IQR 0.6–1.0; range 0.3–1.9) mg/kg/day, and 0.7 (IQR 0.5–0.9; range 0.4–1.6) mg/kg/day in the non-macrolide + steroid group. Only prednisolone and methylprednisolone were used in the patients.

### 3.2. Treatment Response

Of the 190 patients with lobar or segmental pneumonia, only 16.8% (*n* = 32/190) were responsive to the initial macrolide monotherapy, whereas 83.2% (*n* = 158/190) of the patients were refractory to initial macrolide therapy.

**Figure 2:**
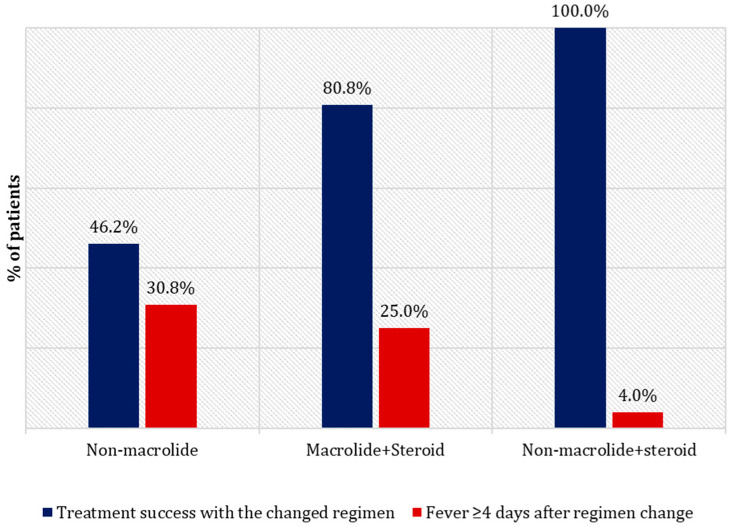
Comparison of the percentage of patients with treatment success versus fever duration of 4 days or more after regimen change. The treatment success rates of the regimens were as follows: non-macrolide, 46.2%; macrolide + steroid, 80.8%; non-macrolide + steroid, 100.0%. The percentage of patients with a fever duration of ≥4 days was highest in the non-macrolide group (30.8%) and lowest in the non-macrolide + steroid group (4.0%).

The overall treatment success rates of the regimens were 46.2%, 80.8%, and 100.0% in the non-macrolide, macrolide + steroid, and non-macrolide + steroid groups, respectively. The percentage of patients with a fever duration of ≥4 days was highest in the non-macrolide group (30.8%) and lowest in the non-macrolide + steroid group (4.0%) (Figure 2). Moreover, 53.8% of the patients in the non-macrolide group eventually needed an addition of steroids, whereas 19.2% of the patients in the macrolide + steroid group needed a change in antibiotic to a non-macrolide before fever resolution.

Patients in the non-macrolide + steroid group had the shortest duration of fever after a regimen change of 1 (IQR, 0–3) day compared with patients in the non-macrolide group and macrolide + steroid group, who had a fever duration of 2 (IQR 1–4) days and 2 (IQR 1–3.3) days (*p* = 0.004) after regimen change, respectively (Table 1). When the outcomes of patients who initially switched to a non-macrolide were compared with patients who switched to a non-macrolide + steroid, those who were administered only a non-macrolide had an odds ratio (OR) of 10.7 (95% confidence interval [CI], 1.5–108.7; *p* = 0.046) higher likelihood of a fever duration ≥4 days after the change in regimen. Patients who switched to macrolide + steroid were 8.0 (95% CI, 1.3–61.7; *p* = 0.046) times more likely to have a fever ≥4 days after the switch, compared with patients who switched to non-macrolide plus steroid.

### 3.3. Changes in Laboratory Markers between the Groups

Changes in initial and follow-up laboratory markers according to treatment groups are shown in Table 2. All three groups showed a decrease in CRP: 2.0 mg/dL to 1.6 mg/dL in the non-macrolide group (*p* = 0.015); 3.2 mg/dL to 1.9 mg/dL in the macrolide + steroid group; and 2.5 mg/dL to 1.5 mg/dL in the non-macrolide + steroid group (*p* = 0.023). All three groups had high LDH at both initial and follow-up (Table 2).

### 3.4. Factors Associated with Fever Duration after Admission

Univariable analysis was carried out to assess factors associated with fever duration after admission. Because fever duration is generally associated with the extent of pneumonia, the degree of initial and improving inflammation, and the appropriate treatment regimen, chest X-ray findings, laboratory markers, and treatment regimen were all included in the univariate analyses. In the linear regression model, elevated follow-up CRP (ß, 0.246; 95% CI, 0.110–0.382; *p* = 0.001), AST (ß, 0.014; 95% CI, 0.006–0.021; *p* < 0.001), and ALT (ß, 0.007; 95% CI, 0–0.014; *p* = 0.037) levels were shown to be associated with an increase in fever duration. Both macrolide + steroid (ß, −1.704; 95% CI, −2.310–−1.099; *p* < 0.001) and non-macrolide + steroid therapy (ß, −2.452; 95% CI, −3.260–−1.645; *p* < 0.001) were associated with a decrease in the duration of fever after admission. A multivariable analysis was undertaken to find factors significantly associated with the fever duration after admission after adjusting for potentially confounding variables. All factors with statistical significance (*p* < 0.050) in the univariable analyses were included in the multivariable analyses. Elevated follow-up CRP (ß, 0.169; 95% CI, 0.050–0.287; *p* = 0.006) was associated with a longer fever duration, whereas macrolide + steroid therapy (ß, −1.694; 95% CI, −2.463–−0.925; *p* < 0.001), and non-macrolide + steroid therapy (ß, −2.224; 95% CI, −3.321–−1.127; *p* < 0.001) were shown to be significantly associated with a shorter fever duration after admission (Table 3).

## 4. Discussion

*Mycoplasma pneumoniae* is the most common cause of bacterial community-acquired pneumonia during childhood [12]. MRMP has been increasing globally, and a study in Korea showed that in 2000, 0% of MP cultured from children with Mycoplasma infections was found to carry macrolide resistance genes. However, the proportion increased steadily to 62.9% in 2011 [13]. In 2015, another study in Korea showed that the proportion of MRMP increased to 87.2% [14]. In Japan, a study undertaken during 2010–2011 showed that the rate of MRMP was 89.2%, and children with MRMP infections showed a longer fever resolution time [15]. With the increase in the proportion of MRMP causing infections in children, delayed effective antimicrobial treatment can lead to the progression of the disease [16,17]. Therefore, in children refractory to initial macrolide therapy, a few treatment options have been recommended, such as the addition of steroids or a switch to a non-macrolide [10,18,19]. However, due to the potential toxicities of tetracyclines, such as teeth discoloration and enamel hypoplasia in children under 8 years old [20], and irreversible arthropathies of fluoroquinolones in children [21], there are concerns about using these two classes of antibiotics in children. Thus, these two drug classes should only be used in limited situations where the benefits outweigh the risks, which is the case for severe pneumonia caused by MRMP. However, to date, there is still a lack of data on which alternative treatment is superior.

In this study, we found that of the 190 patients admitted for the treatment of lobar or segmental MP pneumonia, 83.2% were refractory to initial macrolide therapy and had persistent fever without improvement in pneumonic lesions on chest radiographs after at least 3 days of administration. Because macrolide resistance was unable to be tested in this study, the percentage of children who are refractory to initial macrolide therapy is similar to that of a meta-analysis of global trends in the proportion of MRMP infections. This analysis reported that the worldwide proportion of MRMP infections increased to 76.5% in 2019 [22].

Failure to respond to initial macrolide treatment in patients included in this study led to the decision to switch their treatment regimen in accordance with the 2019 KSPID and KAPARD guidelines [10]. These guidelines recommend a switch to a non-macrolide or the addition of steroids in combination with a macrolide or non-macrolide for patients who have severe pneumonia caused by MP and are refractory to macrolide 72 h after initiation. In our study, it was found that the non-macrolide + steroid group had the highest treatment success rate of 100%, achieving defervescence and improvement in symptoms, or decreasing infiltrations on chest X-rays. Comparatively, those who switched to a non-macrolide had a 10.7-times higher risk of prolonged fever, and those who switched to macrolide + steroid had an 8.0-times higher risk of prolonged fever of 4 days or more.

When comparing the treatment responses of patients who were given a macrolide + steroid versus non-macrolide + steroid, a multivariate logistic regression analysis showed that both were significant factors associated with a shorter fever duration after admission in children with macrolide-refractory MP pneumonia. However, the ß coefficient for the non-macrolide + steroid group had a greater degree of negativity compared to the non-macrolide + steroid group (ß, −2.452; 95% CI, −3.260–−1.645 vs. −1.704; 95% CI, −2.310–−1.099; respectively), showing that children treated with non-macrolide + steroid had a faster fever resolution time after admission compared to those treated with macrolide + steroid. Although the mechanisms underlying the hyperinflammatory and cytokine responses following lung injury from MP infections are not yet clearly understood, many studies have shown that the use of steroids has been effective in reducing lung injury caused by MP [12,23,24,25], especially in macrolide refractory cases [26]. However, data from our study show that in some cases, the addition of steroids to a macrolide was not enough to control the infection. In fact, 19.2% of the patients in the macrolide + steroid group needed a change to non-macrolide + steroid in order to achieve fever resolution. This emphasizes the importance of microbiologic resolution to control severe infections.

In this study, the extent of pulmonary infiltrations on chest X-rays, such as bilateral involvement, lobar versus segmental consolidations, the presence of pleural effusion, and atelectasis, did not show significant associations with fever duration after admission. The degree of chest X-ray abnormalities usually reflects disease severity [27]; however, this was not the case in our study. A possible explanation for this is that the majority of the patients included in this study had a similar extent of pneumonia. The degree of pneumonic infiltrations could not be represented numerically; therefore, a limitation existed in using only the descriptive findings to portray the extent of pneumonia. On the contrary, elevated CRP levels at follow-up were shown to be significantly associated with a longer fever duration after admission. This shows that CRP levels can be a useful marker in determining the degree of remaining inflammation in mycoplasma infections, similar to other bacterial infections [28].

There were several limitations in this study. Due to its retrospective nature, there may have been selection bias. However, our data show that statistically, the three treatment groups were uniform in demographic, clinical presentations, and disease severity, allowing adequate comparisons of therapeutic responses to the three different treatment regimens. In addition, the number of patients included in the non-macrolide group was small, and data from further studies are needed to support the findings of our study. In addition, of the antibiotics within the macrolide class, only roxithromycin and clarithromycin were used in this study, and azithromycin was not prescribed due to extensive distribution into tissues and longer elimination half-life. Therefore, caution is needed when applying the results of the studies to all macrolides [29].

## 5. Conclusions

To conclude, in patients with lobar or segmental MP pneumonia that were refractory to initial macrolide therapy, a non-macrolide antibiotic and steroid combination had the highest treatment success rate and shortest duration of fever. Therefore, in children who present with severe or critical MP pneumonia and are unresponsive to initial macrolide therapy, a non-macrolide antibiotic and steroid may be appropriate for fever resolution and treatment success.

## Figures and Tables

**Table 1:** Demographic and laboratory data according to treatment groups of patients who were refractory to initial macrolide monotherapy.

	Total N = 158		Non-Macrolide *n* = 13	Macrolide + Steroid *n* = 120	Non-Macrolide + Steroid *n* = 25	*p*
Age, years, median (IQR)	7 (5–9)		7 (6–11)	7 (5–9)	6 (4–9)	*0.610*
Male, no. (%)	73 (46.2)		3 (23.1)	58 (48.3)	12 (48.0)	*0.218*
Fever duration before admission, days, median (IQR)	5 (4–6)		5 (5–6)	5 (3–6)	5 (4–7)	*0.374*
Fever duration after regimen change, days, median (IQR)	2 (1–3)		2 (1–4)	2 (1–3.3)	1 (0–3)	*0.004*
Admission duration, days, median (IQR)	5 (4–6)		4 (3–5.5)	5 (4–6)	5 (3–5.5)	*0.010*
Bilateral lung involvement, no. (%)	29 (18.4)		3 (23.1)	20 (16.7)	6 (24.0)	*0.621*
Lobar pneumonia, no. (%)	62 (39.2)		6 (46.1)	47 (39.2)	9 (36.0)	*0.831*
Pleural effusion, no. (%)	15 (9.5)		0	15 (12.5)	0	–
Atelectasis, no. (%)	1 (0.6)		0	1 (0.8)	0	–

IQR, interquartile range. Macrolide—roxithromycin or clarithromycin; non-macrolide—doxycycline or levofloxacin.

**Table 2:** Changes in laboratory parameters according to treatment groups.

	Non-Macrolide *n* = 13		*p*	Macrolide + Steroid *n* = 120		*p*	Non-Macrolide + Steroid *n* = 25		*p*
	Initial	Follow up		Initial	Follow up		Initial	Follow up	
WBC, 10^6^/uL	7120 (5960–8350)	8455 (5493–9850)	*0.580*	6730 (5745–9118)	7685 (5745–9640)	*0.281*	6720 (5960–10,050)	7515 (6360–8335)	*0.420*
ESR, mm/h	21.0 (17.0–26.0)	18.0 (14.0–24.0)	*0.263*	23.0 (17.0–31.0)	28.0 (18.5–33.5)	*0.581*	25.0 (18.8–31.0)	18.0 (11.0–22.0)	*0.128*
CRP, mg/dL	2.0 (1.6–5.2)	1.6 (1.2–2.9)	*0.015*	3.2 (1.3–4.8)	1.9 (0.7–3.8)	*<0.001*	2.5 (1.8–6.4)	1.5 (1.1–2.7)	*0.023*
LDH, mg/dL	590.0 (535.0–663.5)	781 (591.0–819.0)	*0.073*	658.5 (550.0–810.0)	714.0 (599.0–844.0)	*0.130*	649.0 (559.0–1031.5)	626.5 (610.5–862.3)	*0.238*
AST, mg/dL	35.0 (27.0–39.0)	33.0 (33.0–42.0)	*0.742*	34.0 (28.0–42.0)	33.0 (27.3–39.8)	*0.401*	29.0 (27.0–44.0)	35.5 (23.8–44.0)	*0.414*
ALT, mg/dL	15.0 (13.0–28.0)	28.5 (15.3–41.8)	*0.591*	15.0 (13.0–20.3)	19.0 (15.0–29.8)	*0.012*	16.0 (11.0–23.0)	45.5 (17.5–50.5)	*0.357*

Values are median (interquartile range). Abbreviations: ALT—alanine transaminase; AST—aspartate aminotransferase; CRP—C-reactive protein; LDH—lactate dehydrogenase. Macrolide—roxithromycin or clarithromycin; non-macrolide—doxycycline or levofloxacin.

**Table 3:** Factors associated with fever duration after admission in children with macrolide-refractory *Mycoplasma pneumoniae* pneumonia.

	Univariable					Multivariable				
	ß	HR (95% CI)		*SE*	*p*	ß	HR (95% CI)		*SE*	*p*
		Lower	*Upper*				Lower	*Upper*		
Age	−0.080	−0.171	0.010	0.046	*0.080*					
Chest X-ray image findings										
Bilateral lung involvement	0.059	−0.66	0.778	0.364	*0.871*					
Lobar pneumonia	−0.389	−0.955	0.178	0.287	*0.177*					
Pleural effusion	0.647	−0.223	1.517	0.440	*0.144*					
Atelectasis	2.478	−1.010	5.965	1.765	*0.162*					
Initial laboratory findings										
CRP	0.059	−0.018	0.137	0.039	*0.134*					
AST	0.018	0	0.036	0.009	*0.053*					
ALT	0.006	−0.008	0.020	0.007	*0.398*					
Ferritin	0.001	−0.008	0.01	0.003	*0.805*					
Follow-up laboratory findings										
CRP	0.246	0.110	0.382	0.068	*0.001*	0.169	0.050	0.287	0.060	*0.006*
AST	0.014	0.006	0.021	0.004	*<0.001*	0.008	−0.003	0.018	0.005	*0.151*
ALT	0.007	0	0.014	0.003	*0.037*	0.002	−0.009	0.012	0.005	*0.741*
Treatment regimen										
Non-macrolide	R									
Macrolide + steroid	−1.704	−2.310	−1.099	0.306	*<0.001*	−1.694	−2.463	−0.925	0.386	*<0.001*
Non-macrolide + steroid	−2.452	−3.260	−1.645	0.409	*<0.001*	−2.224	−3.321	−1.127	0.551	*<0.001*

Abbreviations: ALT—alanine transaminase; AST—aspartate aminotransferase; CI—confidence interval; CRP—C-reactive protein; HR—hazard ratio; linear regression model, dependent variable: duration of fever after admission. Macrolide—roxithromycin or clarithromycin; mon-macrolide—doxycycline or levofloxacin.

## Data Availability

Data are partially available upon request to the corresponding author.
